# Health system barriers to vaccination among Ukrainian economic migrants in Poland

**DOI:** 10.1093/eurpub/ckac129.185

**Published:** 2022-10-25

**Authors:** M Gańczak, P Kalinowski, M Kowalska

**Affiliations:** 1 Department of Infectious Diseases, University of Zielona Gora, Zielona Gora, Poland; 2 Department of Hygiene and Epidemiology, Medical University Lublin, Lublin, Poland

## Abstract

**Background:**

Before Russia's aggression, Ukrainians were the largest migrant group in Poland. However, data on health system barriers to vaccination in this vulnerable group were not collected. The study aimed to explore barriers to child MMR/HPV vaccination and related access to Polish healthcare services.

**Methods:**

Between December 2021-January 2022, a qualitative study of Ukrainian migrants (UM) living in Poland, recruited through a snowball sampling method, was conducted as a part of RIVER-EU project. 8 focus groups were held with 49 UM aged 16-44 years, followed by interviews with 12 health care providers (HCP).

**Results:**

UM and HCP experienced communication barriers despite language similarities. HCP reported that since a UM is not willing to register at a GP practice he cannot be reached by the Polish vaccination system. UM experienced challenges in navigating the system and accessing credible information in Ukrainian, no official local health authority vaccination material existed either. UM complained that there are no translated versions of vaccination materials accessible at the PHC facilities and they are not adequately informed about the possible side effects of vaccines; HCP reported the lack of time provided by the system for health promotion. UM were not familiar and rather hesitant regarding self-paid vaccines, such as HPV vaccine. In Poland and Ukraine this topic is not targeted at school curriculum neither by information campaigns. UM reported that HPV vaccine is not of interest for them due to the high cost, however they might consider it for their daughters, if the cost was fully refunded.

**Conclusions:**

The study identified main health system barriers to child vaccination regarding economic UM in Poland, seen from the perspective of migrants and HCP and pinpointed issues for improvement. This can serve as a starting point to confront vaccination related challenges in the context of Ukrainian refugee crisis Poland is currently dealing with.

